# MetaboAnalystR 2.0: From Raw Spectra to Biological Insights

**DOI:** 10.3390/metabo9030057

**Published:** 2019-03-22

**Authors:** Jasmine Chong, Mai Yamamoto, Jianguo Xia

**Affiliations:** 1 Institute of Parasitology, McGill University, Montreal, QC H3A 0G4, Canada; jasmine.chong@mail.mcgill.ca (J.C.); mai.yamamoto@mail.mcgill.ca (M.Y.); 2 Department of Animal Science, McGill University, Montreal, QC H3A 0G4, Canada

**Keywords:** global metabolomics, LC-MS, spectra processing, pathway analysis, enrichment analysis

## Abstract

Global metabolomics based on high-resolution liquid chromatography mass spectrometry (LC-MS) has been increasingly employed in recent large-scale multi-omics studies. Processing and interpretation of these complex metabolomics datasets have become a key challenge in current computational metabolomics. Here, we introduce MetaboAnalystR 2.0 for comprehensive LC-MS data processing, statistical analysis, and functional interpretation. Compared to the previous version, this new release seamlessly integrates XCMS and CAMERA to support raw spectral processing and peak annotation, and also features high-performance implementations of mummichog and GSEA approaches for predictions of pathway activities. The application and utility of the MetaboAnalystR 2.0 workflow were demonstrated using a synthetic benchmark dataset and a clinical dataset. In summary, MetaboAnalystR 2.0 offers a unified and flexible workflow that enables end-to-end analysis of LC-MS metabolomics data within the open-source R environment.

## 1. Introduction

Metabolomics is the comprehensive study of all small molecule metabolites (<1500 Da) detected within a biological system. An individual’s metabolic profile represents the functional product of interactions among genetics, lifestyle, environment, diet, and native microbiota, which closely reflects his or her health status [1,2]. The metabolome thus serves as the link between genotype and phenotype, and metabolomics will play a critical role in the development and implementation of precision medicine [3,4].

**Figure 1:**
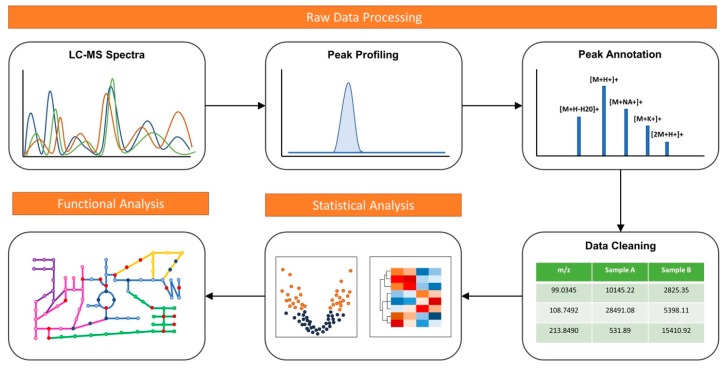
A typical metabolomics data analysis workflow including raw data processing, statistical analysis and functional interpretation.

There are two general approaches in conducting metabolomics. Targeted metabolomics aim to study a predefined set of metabolites, requiring familiarity with the system [3]. Untargeted metabolomics, also known as global metabolomics, aim to measure the global set of metabolites within a sample without a prior knowledge of the system. A typical metabolomics analysis workflow involves three main steps: raw data processing, statistical analysis, and functional interpretation (Figure 1). Global metabolomics requires more sensitive analytics platforms to achieve comprehensive measurement. High-resolution liquid chromatography-mass spectrometry (LC-MS) systems is currently the main workhorse for global metabolomics. The platform often generates thousands of signals, including true biological signals from metabolites, their adducts, fragments, and isotopes, as well as noise signals from contaminants and artifacts [5]. Computational tools able to significantly reduce noise in MS spectra are crucial for more meaningful downstream analyses [6].

There are several powerful computational workflows including commercial tools such as Mass Profiler (Agilent Technologies) and Compound Discoverer (Thermo Scientific), cloud-based software such as XCMS Online [7] and Workflow4Metabolomics [8], desktop software such as MZmine2 [9], MS-DIAL [10], and Open-MS [11], and finally R packages such as MAIT [12] and metaX [13]. Most of these software focus on addressing one of the two main tasks: spectral processing or statistical analysis. Consequently, users must often learn several tools to meet their data analysis needs. Due to compatibility issues, users often have to write scripts to convert outputs from one tool in order to use another tool. 

Tools for functional interpretation of global metabolomics data is in general lacking or poorly addressed [14,15]. A prerequisite for metabolomics data interpretation is metabolite identification, thereby permitting the contextualization of annotated peaks in metabolic pathways and their integration with other omics data. However, even with high mass accuracy afforded by the current high-resolution MS platforms, it is often impossible to uniquely identify a given peak based on its mass alone [16]. Researchers usually need to manually search compound databases and then perform further experimental validations such as tandem MS. Novel bioinformatics tools are urgently needed to enable researchers to gain biological insights with a minimum amount of manual efforts. To get around this bottleneck, a key concept is to shift the unit of analysis from individual compounds to individual pathways or a group of functionally related compounds (i.e., metabolite sets [17]). The general assumption is that the collective behavior of a group is more robust against a certain degree of random errors of individuals. The mummichog algorithm is the first implementation of this concept to infer pathway activities from a ranked MS peaks [18]. The original algorithm implements an over-representation analysis (ORA) method to evaluate pathway-level enrichment based on significant peaks. An alternative approach is the Gene Set Enrichment Analysis (GSEA) method, which is widely used to test enriched functions from ranked gene lists [19]. Unlike ORA, GSEA considers the overall ranks of features without using a significance cutoff. It can detect subtle and consistent changes which could be missed from using ORA methods. Despite its widespread applications in gene expression profiling, it has not yet been applied to global metabolomics. 

MetaboAnalyst is one of the most widely used tools for statistical and functional analysis of metabolomics data [20,21,22,23]. It was initially designed for targeted metabolomics, and subsequent releases gradually introduced many statistical methods applicable to both targeted and untargeted metabolomics. Due to its web-based implementation, there is very limited support for raw spectra processing and peak annotation. The most recent update (version 4.0) was released with a companion R package, MetaboAnalystR (v1.0), to help tackle issues associated with workflow customization, reproducibility, and handling large datasets [24]. 

Here, we present MetaboAnalystR (v2.0) to address the two important gaps left in its previous version: (1) raw spectral processing - we have implemented comprehensive support for raw LC-MS spectral data processing including peak picking, peak alignment, and peak annotations; and (2) functional interpretation directly from *m*/*z* peaks - in addition to an efficient implementation of the mummichog algorithm [18], we have added a new method to support pathway activity prediction based on the well-established GSEA algorithm [19]. We showcase the performance of these new functions through two case studies.

## 2. Results

MetaboAnalystR 2.0 consists of a series of flexible R functions that can take a variety of user-supplied data and parameters to perform end-to-end metabolomics data analysis. The source code is freely available at the GitHub repository (https://github.com/xia-lab/MetaboAnalystR). Detailed instructions, tutorials, troubleshooting tips, example datasets, and analyses discussed in this paper are also available in this repository. 

To demonstrate the utility of MetaboAnalystR 2.0 workflow, we present the results from two case studies: (i) a synthetic benchmark dataset to evaluate the raw MS spectra processing functions, with a focus on its peak detection and quantification performance; and (ii) a clinical pediatric inflammatory bowel disease (IBD) dataset to showcase the overall workflow, with a focus on its capacity to provide biological insights. All R scripts to perform the entire metabolomics data analysis pipeline are available from the MetaboAnalystR GitHub repository under the section “Case Studies”. The accompanying vignette (“The MetaboAnalystR 2.0 Workflow”) provides a step-by-step tutorial to demonstrate how to use MetaboAnalystR 2.0 to perform an end-to-end metabolomics data analysis on a subset of 12 of the 48 clinical IBD samples. This tutorial was created on a Dell XPS 9570 laptop running Ubuntu 16.04 with 16 GB of memory. The total running time of the tutorial was 14 min, averaging ~1.25 min per sample, using 6 cores in parallel and 10.5 GB of memory.

### 2.1. Benchmark Case Study

We first demonstrate the accuracy of the raw data preprocessing module using a benchmark dataset comprised of a mixture of 1100 known compounds ranging in size from 100 to 1300 Da [25]. The original study used a targeted analysis to obtain their benchmark feature list, which we used as the ground truth to evaluate our workflow. As shown in Table 1, the original study detected 35,215 peaks using XCMS Online, with 820 classified as true features. Using the same data preprocessing parameters as published, MetaboAnalystR 2.0 detected 21,013 peaks from the benchmark data. Among them, 732 matched the true features based on *m*/*z* and retention time (10 ppm and 0.3 min RT tolerance). Next, we compared the number of accurately quantified true features using MetaboAnalystR 2.0 to those from the original manuscript using XCMS Online (Table 1). Features were accurately quantified if their fold changes had a <20% relative error as compared to the benchmark data. MetaboAnalystR 2.0 accurately quantified 632 features and identified 45 truly discriminating features.

### 2.2. IBD Case Study

The 48 fecal samples were obtained from 24 pediatric Crohn’s Disease (CD) patients and 24 pediatric healthy controls (Table S1). Our workflow detected 8187 features which were further reduced to 6930 features after filtering out isotopes and features missing in >50% of samples. After exclusion of low-variance features, a total of 4113 features were analyzed using the standard MetaboAnalystR functions.

**Figure 2:**
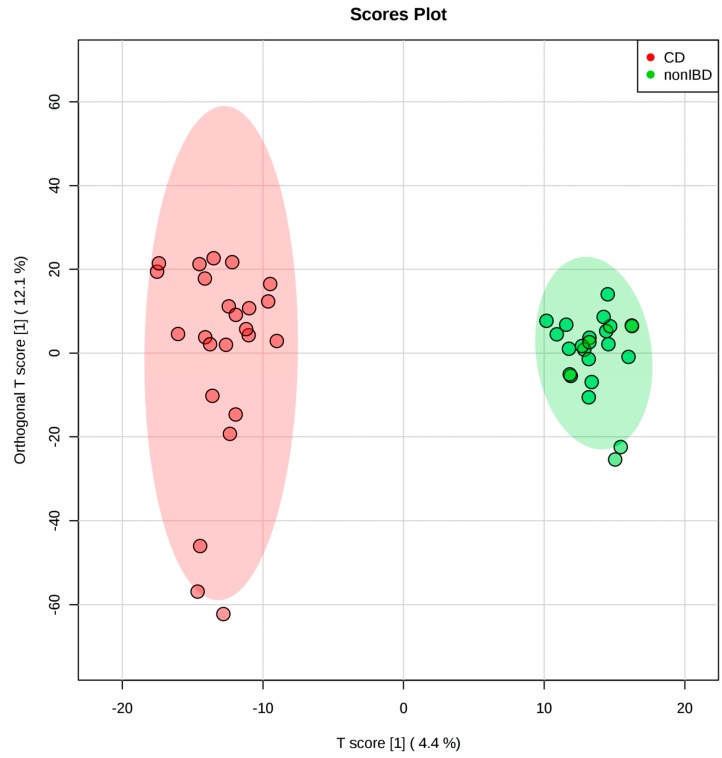
The OPLS-DA score plot based on the stool metabolome of 24 pediatric Crohn’s disease patients and 24 healthy children

Mann–Whitney U test and fold change analysis detected 59 features that were significantly different between CD and healthy controls. Differences between CD and healthy controls were evaluated using PCA, PLS-DA, and OPLS-DA. The PCA showed an overlapping of clusters along the first two components, with CD exhibiting a wider data distribution (Figure S1). This indicates an overall similarity of the metabolic profiles between CD and healthy controls but larger heterogeneity within CD patients. The PLS-DA score plot showed a clear separation between the two groups (Figure S2). Ten-fold cross validation of two PLS-DA components gave an *R2* of 0.912 and *Q2* of 0.424 (Figure S3). The OPLS-DA score plot shows a clear separation between CD and healthy controls (Figure 2) with an R2Y of 0.979 and Q2 of 0.522, respectively. To further evaluate the model, we performed permutation tests (*n* = 1000). The empirical *p* values were 0.026 for R2Y and <0.001 for Q2. Altogether, a clear distinction between the metabolome of CD and healthy controls was observed.

**Figure 3:**
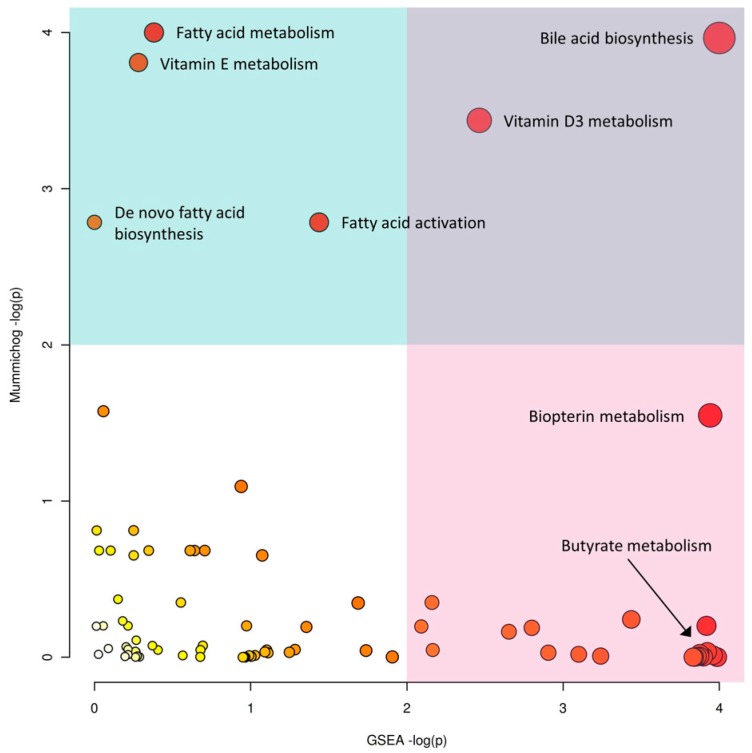
The scatter plot integrating GSEA (x-axis) and mummichog (y-axis) pathway analysis results. The size and color of the circles correspond to their transformed combined *p*-values. The blue and pink areas highlight significant pathways based on either GSEA (pink) or mummichog (blue).

To gain potential biological insights from the global metabolomics data, we applied both mummichog and GSEA algorithms and integrated their results (Figure 3). Mummichog suggested that differentially abundant features between CD and healthy patients were associated with perturbations in bile acid biosynthesis and fatty acid activation, as well as vitamin E, fatty acid, and vitamin D3 metabolism. The GSEA algorithm also identified alterations in bile acid biosynthesis. Moreover, it identified differences in androgen and estrogen biosynthesis and metabolism, squalene and cholesterol biosynthesis, biopterin metabolism, and butyrate metabolism. More details of the top 5 enriched pathways from both methods are given in Table 2.

Interestingly, the GSEA algorithm identified Butyrate metabolism as a significantly enriched pathway, whereas the mummichog algorithm did not. Further inspection (Figure S4) indicated that the mummichog algorithm only utilized the three significant *m*/*z* features to calculate the enrichment score; while GSEA utilized all 20 compound hits (corresponding to 38 *m*/*z* features). Of these features, 145.04962 *m*/*z* was putatively annotated as (S)-2-Aceto-2-hydroxybutanoate (a deprotonated ion), as was 205.07102 *m*/*z* (a formic acid adduct). Furthermore, 124.03917 *m*/*z* corresponded to 2-Butynoate. This demonstrates the ability of GSEA to pick up on subtle changes, such as perturbations in Butyrate metabolism, and the utility of using both algorithms to gain biological insights. 

We further examined the 17 features that overlap between the putatively annotated features in the pathway analysis and the important features found in univariate statistical analysis. Notably, 431.3164 *m*/*z* was putatively annotated as a deprotonated ion of 3-β, 7-α-dihydroxy-5-cholestenoate based on its correspondence to the exact mass of C17336 from the KEGG database [26]. This compound is found in the primary bile acid pathway. Additionally, the same mass also corresponds to a deprotonated ion of 23S, 25, 26-trihydroxyvitamin D3 (CE2202). Exact identification of this feature requires further experiments, which is beyond the scope of this manuscript. In addition to this compound, five additional compounds out of the 17 have been previously found as stool metabolites in the context of IBD [27]. Representative EICs, boxplots, and corresponding information, such as *m*/*z*, retention time, and *p*-values, are highlighted in the Supplemental Materials (Figure S5). 

## 3. Discussion

In this paper, we have described the new functions introduced in MetaboAnalystR 2.0 to support global metabolomics data analysis, covering raw LC-MS spectra processing to generation of biological insights. These functions were showcased through two case studies. 

For the benchmark dataset, despite applying the same parameters used by Li et al. [25], we were unable to reproduce the identification and quantification performance obtained by the original authors using XCMS Online. Their setup detected >14,000 (68%) more features compared to those obtained using our pipeline. We tried several options, including the suggested parameters for a HPLC or UPLC coupled with a Q Exactive HF mass spectrometer. We posit this incongruity arose because the authors did not specify the exact peak width used, which is a critical parameter for peak picking. Additionally, the data conversion step from .RAW to mzML used in our workflow may have resulted in a slight difference in the input data when compared to the data conversion used in XCMS Online. It is also important to note that our workflow integrated the latest version of XCMS (version 3.4.4), which has introduced many new functionalities and updates in existing functions. Overall, our preprocessing workflow performed well, executing peak picking, annotation, and filtering on the eight benchmark samples in less than twenty minutes.

For the IBD case study, we observed a clear separation in the metabolomic profiles between pediatric CD patients and healthy controls using either PLS-DA or OPLS-DA. Furthermore, our analysis highlighted several metabolic pathways associated with CD, without performing accurate metabolite identification. For instance, alterations in bile acid biosynthesis and short-chain fatty acids metabolism are well known among IBD patients [28,29]. Combining the results of pathway analysis and statistical analysis also putatively identified some promising metabolic features that could be used to as potential biomarkers. In addition to bile acids, vitamin D has been shown to play an immunomodulatory role in IBD pathogenesis [30]. Taken together, this use case demonstrates the ease of which MetaboAnalystR 2.0 can be utilized to gain mechanistic insights and generate hypotheses for future experimental validation.

MetaboAnalystR 2.0 has addressed the needs for high throughput raw spectra processing and inferring pathway dysregulation directly from high-resolution MS1 data. A future direction of our workflow includes the integration of MS2 data to support targeted annotations for important peaks assigned to pathways of interest. The function will be developed in coordination with the MetaboAnalyst web server to provide online visual analytics support for molecular networking [31].

## 4. Conclusions

The previous version (v1.0) of MetaboAnalystR features comprehensive normalization and statistical methods inherited from the MetaboAnalyst web server. The version 2.0 not only integrates XCMS and CAMERA to support raw MS spectral processing and peak annotation, but also implements mummichog and GSEA methods for prediction of pathway activities. The performance of this workflow was evaluated on a published benchmark dataset as well as a recent clinical study on IBD. The MetaboAnalystR package is maintained in conjunction with the cloud-based MetaboAnalyst web application and is under continuous development based on the community feedback. Our next focus is on integration with MS2 data as well as development of a Galaxy-based platform for raw data processing [32].

## 5. Materials and Methods

### 5.1. Spectral Processing

Three main wrapper functions have been implemented for metabolomics data processing based on XCMS (version 3.4.4) and CAMERA (version 1.38.1) [33,34,35] including: (i) the *ImportRawMSData* function for reading in raw data files, (ii) the *PerformPeakProfiling* function for peak picking and alignment, and (iii) the *PerformPeakAnnotation* function for annotating isotopes and adducts in processed *m*/*z* data. These functions are described below in further detail.

The *ImportRawMSData* function reads in raw MS data files and saves it as an *OnDiskMSnExp* object. To avoid potential memory issues on a user’s desktop/laptop, the function will limit the number of cores used to half of the available number of cores. The function outputs two plots: the Total Ion Chromatogram (TIC), which provides an overview of the entire spectra, and the Base Peak Chromatogram (BPC), which is a cleaner profile of the spectra based on the most abundant signals. These plots are useful to inform the setting of parameters downstream. For users who wish to view a peak of interest, an Extracted Ion Chromatogram (EIC) can be generated using the *PlotEIC* function. 

The *PerformPeakProfiling* function is a wrapper of several XCMS R functions that performs peak detection, alignment, and grouping in a single step. The resulting peaks are outputted as a *XCMSnExp* object. The function also generates two diagnostic plots including a retention time adjustment map, and a PCA plot showing the overall sample clustering prior to data cleaning and statistical analysis. Users can specify several parameters such as the mass accuracy, peak width, and retention time range using the *SetPeakParam* function to optimize the peak picking function. A detailed table of suggested parameters for common LC-MS platforms is provided in Table S2. 

The *PerformPeakAnnotation* function annotates isotope and adduct peaks using the CAMERA package [35]. CAMERA matches *m*/*z* features to potential isotopes and adducts based on mass using a dynamic rule set. It does not utilize any spectral databases to perform annotation. It outputs the result as a CSV file (“annotated_peaklist.csv”) and saves the annotated peaks as an *xsAnnotate* object. Finally, the peak list is formatted to the correct structure for MetaboAnalystR and filtered based upon user’s specifications using the *FormatPeakList* function. This function permits the filtering of adducts (i.e., removal of all adducts except for [M + H]^+^/[M − H]^−^) and filtering of isotopes (i.e., removal of all isotopes except for monoisotopic peaks). The goal of filtering peaks is to remove degenerative signals and to reduce the file size.

### 5.2. Prediction of Pathway Activities 

Several metabolic databases are supported at the moment including KEGG [26], BioCyc [36], etc. The main mummichog algorithm is available in the *PerformMummichog* function. Users need to specify a pre-defined cutoff based on either t-statistics or fold changes. The *PerformGSEA* function contains the GSEA implementation based on the high-performance *fgsea* R package [37]. 

### 5.3. Benchmark Case Studies

The benchmark data created by Li et al. 2018 [25] is comprised of two standard mixtures (A and B) consisting of 1100 known compounds, with four replicates per mixture. The link to this raw dataset is available in Table S3. For this manuscript, we selected the dataset that was generated from a Q Exactive HF mass spectrometry (Thermo Fisher Scientific) in positive ion mode, coupled with a Dionex UltiMate 3000 HPLC equipped with a ZORBAX Eclipse Plus C18 column (Agilent Technologies). Parameters for our workflow were selected based on the default values provided for HPLC-Q Exactive Orbitrap data on XCMS Online (mass error: 5 ppm and peak width: 10-60 s).

The second dataset consists of pediatric IBD stool samples obtained from the Integrative Human Microbiome Project Consortium (iHMP) [38]. The original study included samples longitudinally collected from IBD patients and non-IBD controls over 50 weeks. The link to this raw dataset is provided in Table S3. For our evaluation purpose, we collected samples that met the following criteria for the diseased group: (i) age between 6 and 19, and (ii) diagnosed with Crohn’s disease. Samples obtained at the earliest clinical visit of each patient who met criteria (i) and (ii) were included in our study. For the healthy control, samples of non-IBD individuals between age 6 and 19 collected during their first and second clinical visits were included. The dataset was generated from a Q-Exactive Plus Orbitrap mass spectrometer (Thermo Fisher Scientific) in negative ion mode, coupled with a Nexera X2-U-HPLC system (Shimadzu Scientific Instruments) equipped with an ACQUITY BEH C18 column (Waters).

All raw data in .RAW format were converted into .mzML format using ProteoWizard 3.0 MSConvert [39] with parameters summarized in the supplemental Materials (Table S4). Following the spectral processing described earlier, data cleaning and statistical analysis were performed on the clinical data using various functions within MetaboAnalystR. Firstly, missing value imputation was performed by replacing them with half of the minimum value found for each feature. Features containing more than 50% missing values across all samples were excluded. Features with nearly constant values across samples were also filtered out based on the inter quantile range (IQR), which removed approximately 25% of total features. Subsequently, value of each feature was normalized with the median value of all features per sample to account for variable water content of stool samples. Finally, generalized log-transformation and auto-scaling were applied to data prior to multivariate statistical analysis. For univariate analysis, non-parametric methods (i.e., Mann–Whitney U test and fold change calculation) were applied to untransformed data to avoid false positives due to data manipulation [40]. A minimum fold change >2 and <0.5, and a false discovery rate (FDR) adjusted *p*-value of 0.05 were used as cut-off values. To infer pathway activities, we applied both mummichog and GSEA to predict pathway activities. The human BiGG and Edinburgh Model (hsa_mfn) library was selected as the pathway database, with the *p*-value cutoff set to 0.05 and the instrumentation accuracy set to 5 ppm. 

## Figures and Tables

**Table 1:** Comparison of peak identification and quantification accuracies using the benchmark dataset between MetaboAnalystR 2.0 and the original manuscript using XCMS Online.

	Methods	Features Detected	True Features		
			Total	Accurately Quantified	Discriminating
Li et al. 2018 [25]	Targeted	-	836	836	-
	Untargeted (XCMS Online)	35215	820	731	45
MetaboAnalystR 2.0	Untargeted	21013	732	632	45

**Table 2:** The top five enriched metabolic pathways identified using the mummichog algorithm (*PerformMummichog*) and GSEA (*PerformGSEA*) in MetaboAnalystR 2.0.

	Mummichog			GSEA	
Pathway Name	Compound Hits *	*p*-Value	Pathway Name	Compound Hits	*p*-Value
Bile acid biosynthesis	29/52	0.00282	Bile acid biosynthesis	52	0.001761
Vitamin E metabolism	20/33	0.00356	Androgen and estrogen biosynthesis and metabolism	10	0.01465
Fatty acid metabolism	9/11	0.00268	Squalene and cholesterol biosynthesis	7	0.02214
Vitamin D3 metabolism	8/10	0.00616	Biopterin metabolism	14	0.07806
Fatty acid activation	10/15	0.01620	Butyrate metabolism	11	0.08318

* The mummichog compound hits represent the number of significant compounds divided by the total number of compound hits per pathway.

## Supplementary Materials

The following are available online at https://www.mdpi.com/2218-1989/9/3/57/s1, Table S1: Characteristics of pediatric IBD patients and healthy controls included in this study; Table S2: Suggested peak picking parameters for commonly used LC-MS platforms; Table S3: Raw datasets used in the Case Studies; Table S4: Parameters used to convert .RAW files to mzML format on ProteoWizard MSConvert; Figure S1: PCA plot of pediatric IBD stool metabolome. Data including 4113 features were median-normalized, log-transformed, and auto-scaled; Figure S2: PLS-DA plot of pediatric IBD stool metabolome. Data including 4113 features were median-normalized, log-transformed, and auto-scaled; Figure S3: Ten-fold cross validation of PLS-DA model (Figure S3) generated from the pediatric IBD stool metabolome data; Figure S4: Boxplots of *m*/*z* features used for functional interpretation; Figure S5: Representative EICs and boxplots of compounds differentially excreted in stool samples of healthy children and pediatric CD patients based on pathway analysis and Mann–Whitney U test (FDR adjusted *p*-value < 0.05).
