# Supplementary material for: MetaboAnalystR 2.0: From Raw Spectra to Biological Insights
Source: Metabolites. 2019 Mar 22;9(3):57. doi: 10.3390/metabo9030057 (PMC6468840; doi:10.3390/metabo9030057)
Supplement: Supplementary file 1 [file metabolites-09-00057-s001.pdf]

Supplemental Information

The MetaboAnalystR 2.0: From Raw Spectra to Biological Insights

Jasmine Chong, Mai Yamamoto, and Jianguo Xia

Table S1. Characteristics of pediatric IBD patients and healthy controls included in this study.

	CD	Healthy
**n**	24	24
**Female gender (n)**	9	15
**Median age, years (range)**	14.5 (8-19)	11 (6-17)

Table S2. Suggested peak picking parameters for commonly used LC-MS platforms.

		SetPeakParam()		
**Vendor**	**Instrument**	**ppm**	**min_pkw**	**max_pkw**
Agilent	HPLC/Q-TOF	30	10	60
Agilent	HPLC/UHD Q-TOF	15	10	60
Agilent	HILIC_HPLC/UHD Q-TOF neg[1]	15	10	120
Bruker	HPLC/Q-TOF neg[1]	10	10	60
Bruker	HPLC/Q-TOF pos[1]	10	5	20
ABSciex	UPLC/TripleTOF	15	5	20
Waters	UPLC/HRMS	15	2	25
Waters	HPLC/TOF	30	10	60
Thermo	UPLC/Q-Exactive	5	5	20
Thermo	HPLC/Orbitrap	3	10	60

[1] neg = negative ion mode, pos = positive ion mode
Parameters are extracted from XCMS Online default settings (PMID 29494574)

Table S3. Raw datasets used in the Case Studies

**Case Study**	**Link**
Benchmark data	https://drive.google.com/drive/folders/1PRDIvihGFgkmErp2fWe41UR2Qs2VY_5G
IBD data	https://ibdmdb.org/tunnel/public/summary.html, under the subheadings HMP2, Metabolites, 2017.23

Table S4. Parameters used to convert .RAW files to mzML format on ProteoWizard MSConvert.

Filter category	Parameter
**Peak picking**	Vendor msLevel = 1 -
**Threshold Peak Filter**	Absolute 1000 most-intense
**Subset**	msLevel 1 – 1

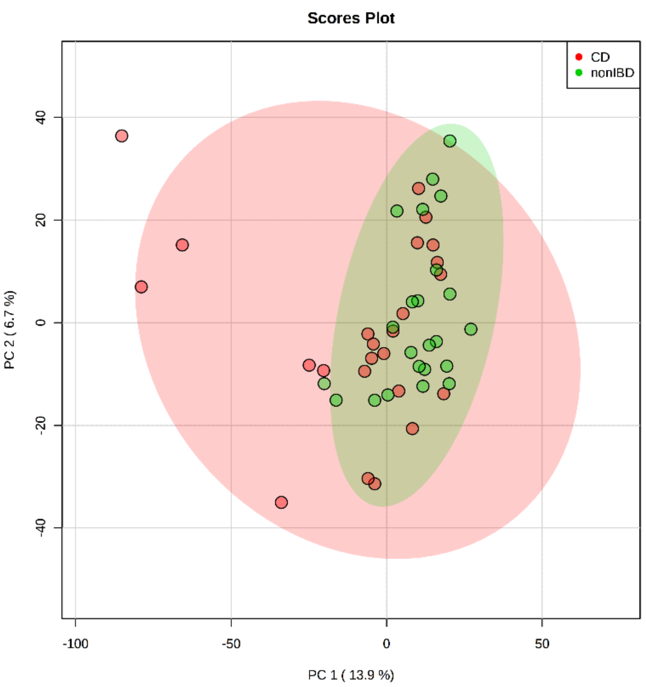

Figure S1. PCA plot of pediatric IBD stool metabolome. Data including 4113 features were median-normalized, log-transformed and auto-scaled.

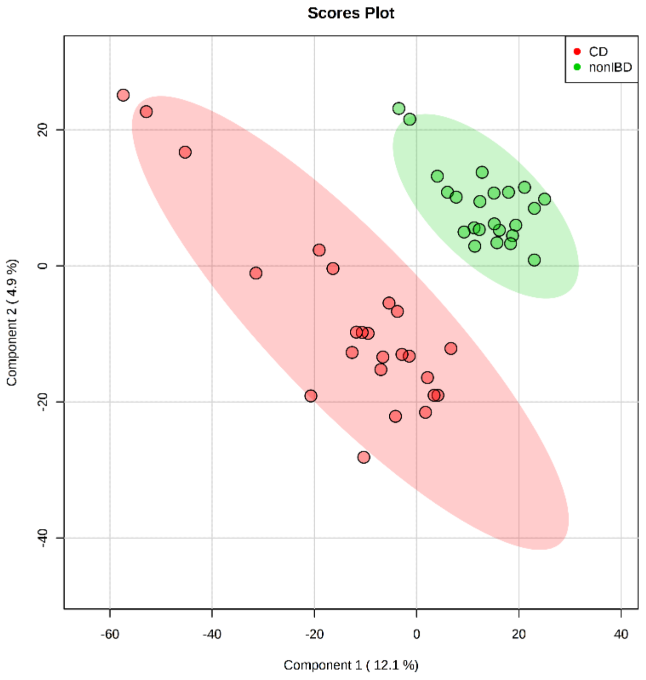

Figure S2. PLS-DA plot of pediatric IBD stool metabolome. Data including 4113 features were median-normalized, log-transformed and auto-scaled.

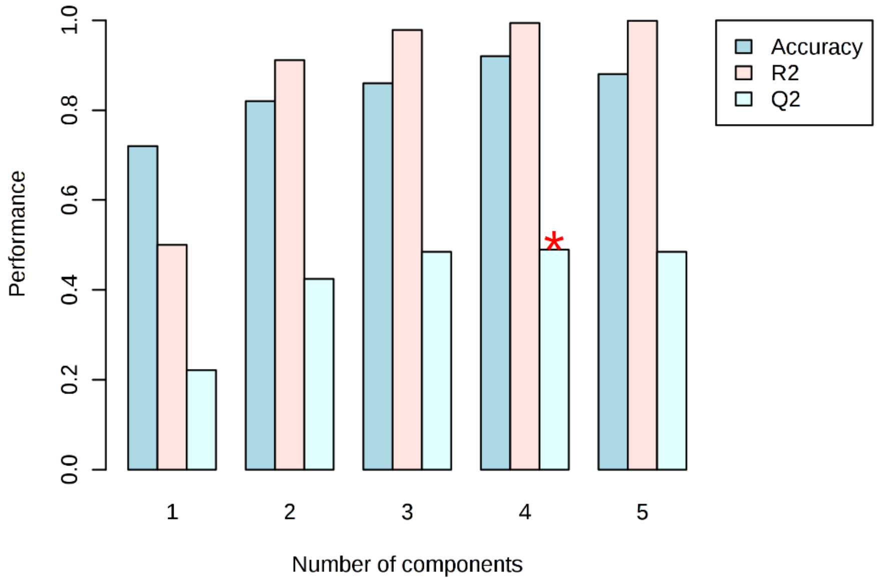

Figure S3. 10-fold cross validation of PLS-DA model (Figure S3) generated from the pediatric IBD stool metabolome data.

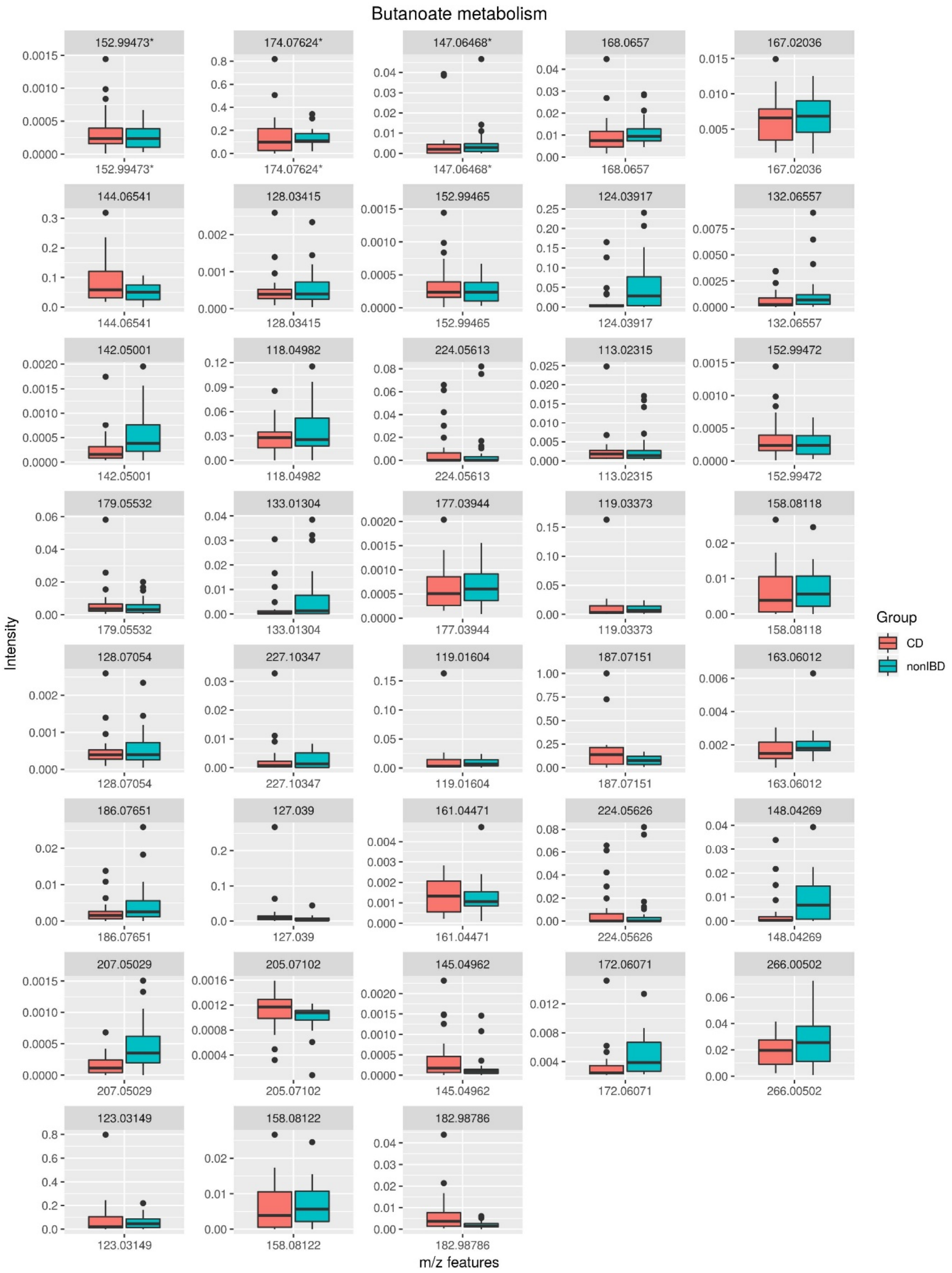

Figure S4. Boxplots of m/z features used for functional interpretation. The m/z features with an asterisk were used by the mummichog algorithm, while all m/z features were used by the GSEA algorithm. The red boxes represent Crohn's disease pediatric patients, and the blue boxes represent healthy patients.

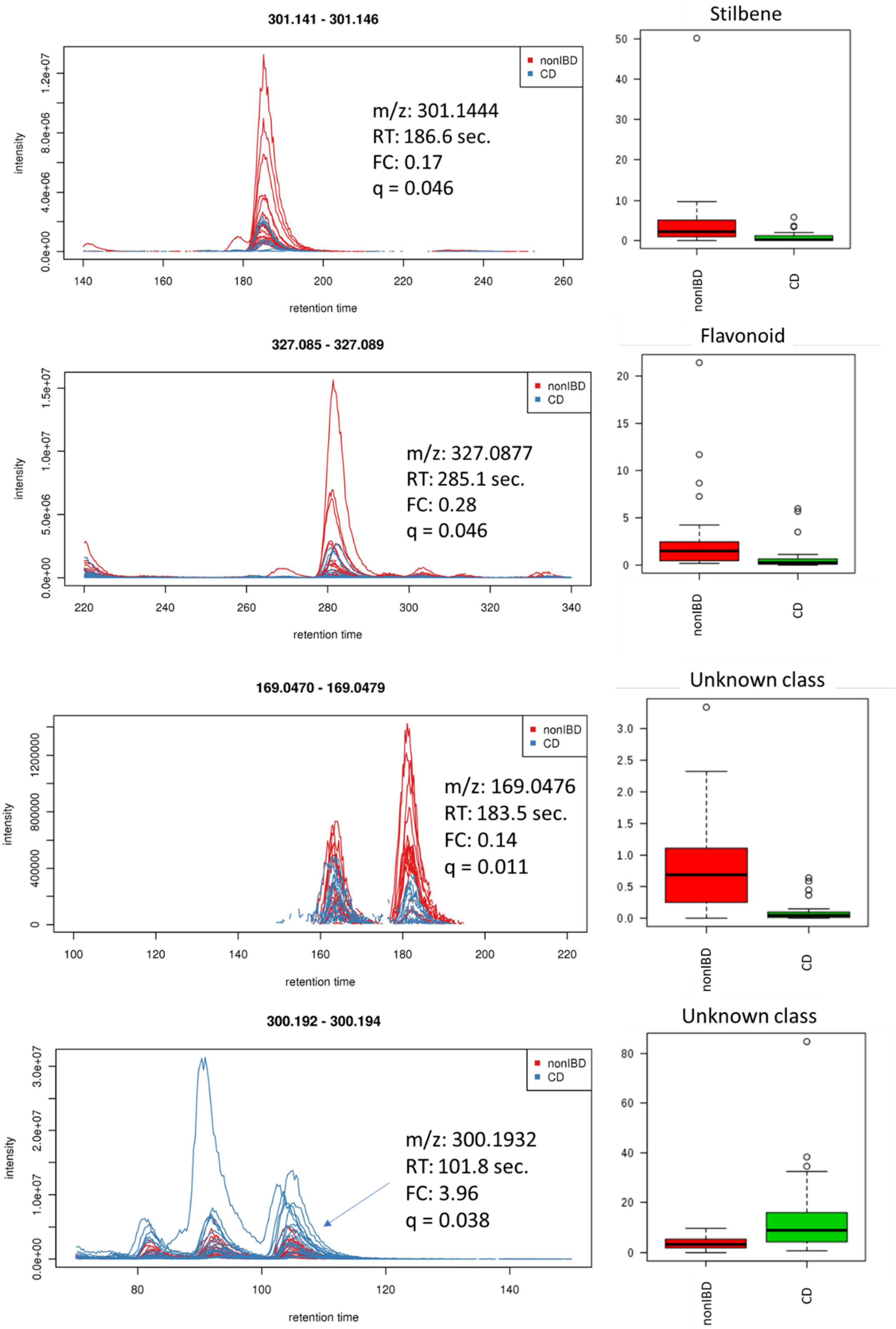
301.141 - 301.146
intensity
0.0e+00
4.0e+06
8.0e+06
1.2e+07
nonIBD
CD
m/z: 301.1444
RT: 186.6 sec.
FC: 0.17
q = 0.046
140
160
180
200
220
240
260
retention time
Stilbene
0
10
20
30
40
50
nonIBD
CD
327.085 - 327.089
intensity
0.0e+00
5.0e+06
1.0e+07
1.5e+07
nonIBD
CD
m/z: 327.0877
RT: 285.1 sec.
FC: 0.28
q = 0.046
220
240
260
280
300
320
340
retention time
Flavonoid
0
5
10
15
20
nonIBD
CD
169.0470 - 169.0479
intensity
0
400000
800000
1200000
nonIBD
CD
m/z: 169.0476
RT: 183.5 sec.
FC: 0.14
q = 0.011
100
120
140
160
180
200
220
retention time
Unknown class
0.0
0.5
1.0
1.5
2.0
2.5
3.0
nonIBD
CD
300.192 - 300.194
intensity
0.0e+00
1.0e+07
2.0e+07
3.0e+07
nonIBD
CD
m/z: 300.1932
RT: 101.8 sec.
FC: 3.96
q = 0.038
80
100
120
140
retention time
Unknown class
0
20
40
60
80
nonIBD
CD

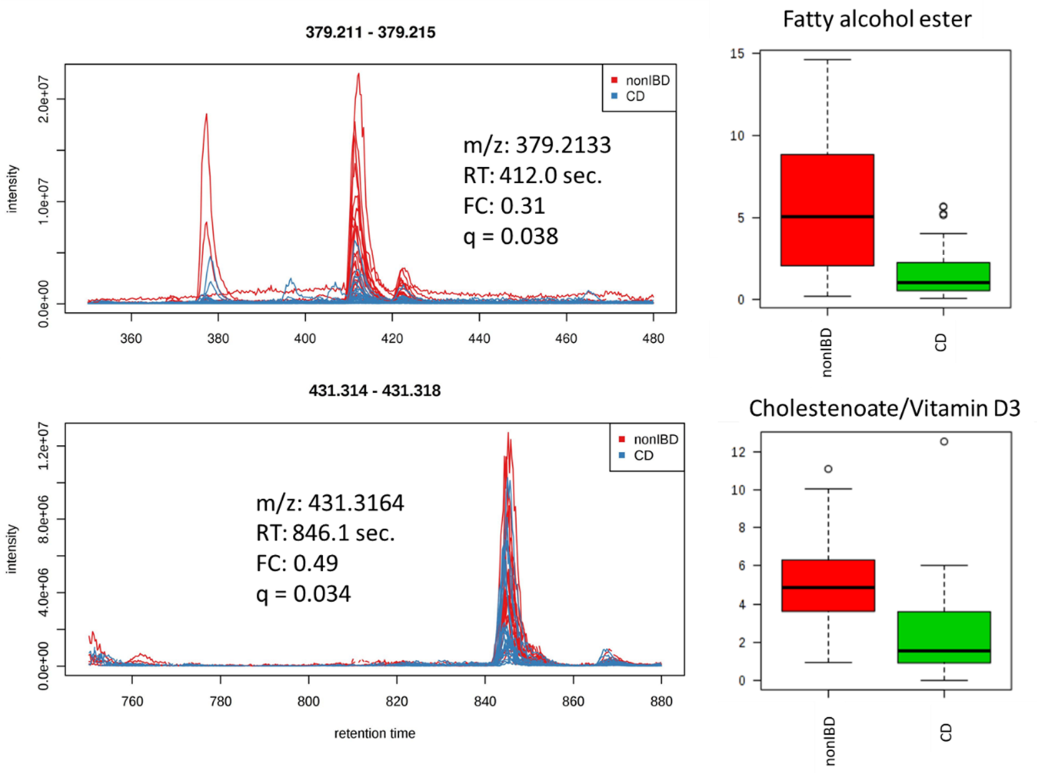

Figure S5. Representative EICs and boxplots of compounds differentially excreted in stool samples of healthy children and pediatric CD patients based on pathway analysis and Mann-Whitney U test (FDR adjusted p-value < 0.05). The m/z of all compounds highlighted above exactly matches with the m/z of compounds detected in the previously published study on adult IBD patients (PMID: 30531976). Putative IDs of each compound assigned in this study are shown above each boxplot.
